# Health and social care managers’ perceptions of implementation of knowledge management in organisations: a secondary qualitative analysis

**DOI:** 10.1108/JHOM-04-2026-0521

**Published:** 2026-10-06

**Authors:** Eevi Karsikas, Merja Meriläinen, Kirsi Koivunen, Outi Kanste

**Affiliations:** Research Unit of Health Sciences and Technology, University of Oulu, Oulu, Finland; Medical Research Center Oulu, Oulu University Hospital and University of Oulu, Oulu, Finland; Oulu University of Applied Sciences, Oulu, Finland

**Keywords:** Health and social care, Knowledge management, Manager, Qualitative study, Secondary qualitative analysis

## Abstract

**Purpose:**

To describe health and social care managers' perceptions of implementation of knowledge management in organisations, using the Managers’ Competence in the Knowledge Management Process Model as a framework. This model comprises five dimensions: anticipating and defining competence, planning competence development and cooperation, developing competence, assessing competence, and managing a culture of competence.

**Design/methodology/approach:**

A secondary qualitative analysis was conducted using existing semi-structured interview data (*n* = 15) collected in spring 2022 from purposive sampling of managers in three public health and social care organisations in Finland and re-analysed using deductive-inductive content analysis.

**Findings:**

Health and social care managers emphasise the importance of systematic, data-based competence anticipation and definition, applied across all career stages and supported by collaborative approaches. They emphasise that strategic planning, competence development, and cooperation are essential, including the documentation of development plans and collaboration with internal and external partners. Competence development is seen as a combination of internal training and the acquisition of external expertise. Competence assessment is considered a vital part of this process, involving systematic evaluation, feedback, communication, and tailored methods for different career stages. Ultimately, managers recognise that establishing a strong culture of competence, led by coaching leadership and strategic change management, is essential for integrating knowledge management into everyday practices.

**Originality/value:**

Health and social care organisations have implemented diverse, structured knowledge management methods that support both organisational learning and the continuous development of staff competencies.

## 1. Introduction

Health and social care organisations increasingly face challenges in retaining competent and experienced staff (Taylor-Clark *et al.*, 2022). In response, it is crucial to recognise human resources as the organisation's most valuable asset (Ayatollahi and Zeraatkar, 2020) and to implement knowledge management systematically as a strategic means of supporting staff competence and organisational performance (Karamitri *et al.*, 2020). Managers play a key role in implementing knowledge management by anticipating and defining competence needs, planning competence development, fostering collaboration, assessing staff competence, and managing a culture of competence. However, the implementation of knowledge management can vary across health and social care organisations (Karsikas *et al.*, 2025a). Managers' perceptions thus provide essential insights into how knowledge management is understood, enacted, and embedded in the daily practices of organisations.

The concept of knowledge management is multifaceted, and research in the field remains fragmented (Dalkir, 2023; Karsikas *et al.*, 2022). Knowledge management is often described as a process (Orzano *et al.*, 2008) that health and social care organisations are encouraged to adopt as a core organisational function (Karamitri *et al.*, 2017). Although these processes have been structured into various frameworks and knowledge management models, the literature identifies multiple ways of organising and managing the development of knowledge and competencies, rather than a single, uniform, or linear process model (Pihlainen *et al.*, 2016; Rahman *et al.*, 2014). For example, Sibbald *et al.* (2016) identify four stages in competence management, identifying, synthesising, utilising, and sharing competence, whereas Popa *et al.* (2018) emphasise knowledge acquisition, sharing, and utilisation alongside employee satisfaction. Similarly, Karamitri *et al.* (2017, 2020) highlight processes such as synthesis, sharing, dissemination, collaboration, and cooperation. The effective implementation of these interrelated processes is particularly critical in health and social care, where knowledge management plays a central role in supporting organisational performance, improving the quality of care, and ensuring efficient use of resources (Karamitri *et al.*, 2020; Kosklin *et al.*, 2022).

In this study, the Manager's Competence in Knowledge Management (MCKM) process model served as the framework. The model comprises five dimensions: anticipation and definition of competence, planning competence development and cooperation, development of competence, assessing competence, and managing a culture of competence (Karsikas *et al.*, 2025a). The first dimension concerns the anticipation and definition of competence, including identifying future competence needs, existing expertise, tacit knowledge, and gaps. This is particularly critical in social and health care services, where workforce shortages are projected to intensify: by 2030, Europe will require millions of additional health professionals (Boniol *et al.*, 2022). Clearly defining competence, therefore, provides a foundation for effective knowledge management by supporting workforce capability development and strengthening service delivery (Søreide *et al.*, 2019).

The second dimension, planning competence development and cooperation, involves strategic, forward-thinking competence planning and the use of anticipated information. It also includes documenting competence plans and integrating cooperation at all stages of knowledge management. Despite its importance for the effective implementation of knowledge management, such planning remains insufficiently embedded in the daily practices of many organisations (Søreide *et al.*, 2019). Effective communication is closely linked to this dimension, as its benefits depend on high-quality relationships and functional cooperation within workers; when cooperation falters, communication becomes less effective, with potential implications for patient care (Wei *et al.*, 2022). The third dimension is the development of competence, which involves the intentional use of competence development methods and practices, along with the recruitment of personnel to ensure the unit's overall competence. Previous research literature indicates that the competence development methods used in social and health care organisations do not fully meet the needs of working life (Sibbald *et al.*, 2016). However, studies have identified ways to better support staff competence development: collaborative work environments have been found to facilitate effective knowledge sharing and continuous learning (Stoumpos *et al.*, 2024), while job rotation may contribute to the integration of temporary staff into permanent roles (Tan *et al.*, 2025).

The fourth dimension, assessing competence, focuses on evaluating personnel skills and abilities. Performance assessment is a key element of knowledge management, supporting the identification of development needs and continuous improvement by evaluating performance, behaviours, and outcomes, as well as providing feedback (Ayatollahi and Zeraatkar, 2020; Gunawan *et al.*, 2019). In social and healthcare settings, competence is most assessed through feedback, while formal performance indicators and systematic appraisal practices are used less frequently, particularly in public-sector organisations (Kiviniitty *et al.*, 2023; Knies *et al.*, 2022). The fifth dimension, managing a competence culture, focuses on fostering support, knowledge sharing, open communication, constructive feedback, and experimentation in competence development. Organisational culture is a key enabler of knowledge management implementation (Ayatollahi and Zeraatkar, 2020): hierarchical cultures tend to hinder knowledge creation and efficiency, whereas non-hierarchical cultures support more active knowledge utilisation and improved organisational performance, particularly in hospital settings (Moreno-Domínguez *et al.*, 2024).

Knowledge management has rarely been studied within the public sector, particularly in health and social care contexts (Moreno-Domínguez *et al.*, 2024; Radević *et al.*, 2023). Despite its clear impact on healthcare finance, management, quality of care, and staff practices, its potential remains underutilised, particularly in healthcare settings (Kosklin *et al.*, 2022). At the same time, earlier research has focused on managerial competencies or developed tools to measure knowledge-sharing behaviour (Karsikas *et al.*, 2024; Karamitri *et al.*, 2020); less attention has been given to how knowledge management is implemented in the organisation.

In Finland, a country of approximately 5.5 million inhabitants, around 530,000 health and social care professionals were employed in 2022, of whom approximately 15,000 held managerial positions (Health and social services personnel, professionals and those trained in the field, 2026; Statistics Finland, 2026). Health and social care services are organised within publicly governed wellbeing services counties undergoing major reforms. The increasing complexity of service integration, workforce management, and organisational change has highlighted the need for systematic knowledge management practices that support evidence-informed decision-making and competence development across organisations (Wellbeing services counties will be responsible for organising health, social and rescue services, 2026). However, challenges remain in implementing these practices and integrating managerial competencies with organisational knowledge processes (Karsikas *et al.*, 2025c). This study addresses that gap by approaching knowledge management as a comprehensive, organisation-wide strategy rather than merely a tool for developing staff competence (Karamitri *et al.*, 2017; Karsikas *et al.*, 2025a; Søreide *et al.*, 2019).

## 2. The study

### 2.1 Aim and research questions

This study aims to explore health and social care managers' perceptions of the implementation of knowledge management in organisations. The objective is to generate insights that support the development of effective knowledge management practices and tools, and to help managers implement high-quality knowledge management in their organisations.

The research question was: What are health and social care managers' perceptions of the implementation of knowledge management in organisations?

### 2.2 Design

This study employed a qualitative descriptive design and involved a secondary qualitative analysis of existing qualitative data. The analysis utilised an existing dataset to address a new research question that differed from the original focus of the primary study (Beck, 2019), thereby maximising the value of participants' contributions through the appropriate reuse of previously collected data (Chatfield, 2020). The original research describes health and social care managers' perceptions of the factors affecting managers' competence in knowledge management (Karsikas *et al.*, 2024). These themes overlap conceptually with the core elements of knowledge management implementation and therefore provide a suitable basis for addressing the current research question, despite the different original focus of data collection. The study followed the Consolidated Criteria for Reporting Qualitative Research (COREQ) guidelines to ensure transparency and rigour in reporting (Tong *et al.*, 2007).

### 2.3 Theoretical framework

The theoretical framework underpinning the authors' understanding of knowledge management implementation in health and social care organisations is based on the MCKM process model (Karsikas *et al.*, 2024b, 2025a). This model comprises five interrelated dimensions: (1) anticipation and definition of competence, (2) planning competence development and cooperation, (3) development of competence, (4) assessment of competence, and (5) management of a competence-oriented culture. Together, these dimensions constitute a comprehensive framework for knowledge management (see Table 1).

**Table 1:** Analysis matrix using the managers' competence in knowledge management instrument Karsikas *et al.* (2025b)

Anticipating and defining competence	Planning competence development and cooperation	Developing competence	Assessing competence	Managing a culture of competence
The manager's competence determines the future competence needs of the staff and anticipates development needs. The competence to decide the core competence, deep competence and supplementary competence of the personnel. Identifies the tacit knowledge and missing competence of the personnel	The managers' competence to plan competence development in a strategy-oriented manner and utilise forecasting information. The competence to draw up a development plan and choose development methods and focus areas, use digital solutions in planning competence development and utilise cooperation in all phases of knowledge management	The managers' competence to utilise competence development methods, guidance methods, cooperation, expanding work and responsibility tasks, study and training, existing competence of personnel, tacit knowledge in competence development, and the recruitment of personnel, ensuring the unit's competence	The manager's competence to assess the factors that promote and hinder the development of the staff's competence, as well as the commitment of The personnel for the development of the competence	Managers' competence to support staff's professional growth, staff information sharing and open communication. The competence motivates to participate in training, inspires to try new methods for skill development and promotes the introduction of sound and operating practices. The competence to give constructive feedback and reward personnel

**Source(s):** The Authors’

### 2.4 Participants and recruitment

The interview dataset used in this study originated from a previous qualitative study. In the primary study, purposive sampling was used to recruit managers with more than five years of managerial experience from two public primary health and social care organisations and one public specialised health and social care organisation in Finland (Polit and Beck, 2017). Managers' contact information was obtained from designated contacts, and respondents were recruited via email. These contacts provided the researchers with the details of 30 managers who met the inclusion criteria. The researchers then contacted these managers individually via email to invite them to participate in the study. Of the 30 managers initially contacted, 15 agreed to participate, while the remaining 15 could not be reached despite repeated attempts. Respondents were sent an interview topic, a fact sheet about the study, a privacy statement, and a consent form along with the interview request. Background information on the participants was collected through a Webropol questionnaire. Interview dates were arranged by email at a mutually convenient time.

### 2.5 Development of the interview guide and data collection

The secondary qualitative analysis used an existing dataset comprising semi-structured interviews (*n* = 15) collected in spring 2022 from managers recruited through purposive sampling in the primary study. The data were re-analysed using deductive-inductive content analysis. The dataset was collected by the first author for the primary study (Karsikas *et al.*, 2024). The researchers had no prior relationship with the respondents. The interviews were conducted via the Microsoft Teams communication platform and recorded with the managers' permission. Field notes were made during the interviews. Prior to the interviews, the managers responded to a Webropol survey to collect demographic information. The semi-structured interview guide was developed in five phases. First, the suitability of semi-structured interviews for the study was assessed. Next, relevant existing knowledge was reviewed to inform the content of the guide. A preliminary version was then created and pilot-tested to ensure clarity and relevance. Based on the pilot results, revisions were made before finalising the complete interview guide (Kallio *et al.*, 2016). The original interview included questions directed at managers concerning both the factors affecting their knowledge management competence and the implementation of knowledge management within the organisation. Table 2 presents the interview questions related to the implementation of knowledge management.

**Table 2:** Semi-structured interview guide

No.	Themes of the interview	Examples of questions
1	Anticipating competence	Could you tell us how you anticipate staff competence in your organisation?
		How could stakeholders be involved in competence anticipation?
2	Defining competence	Could you tell us who has defined staff competence in your organisation?
		How is employee competence defined?
3	Mapping competence	Could you describe how the current state of employee competence is mapped in your organisation?
		What methods are used to map the current state of employee competencies?
4	Developing competence	Could you describe how the development of employee competencies is carried out in your organisation?
		What kind of competence development plan exists in your organisation?
		What methods are used to develop people's competencies?
		How could stakeholders be involved in the development of staff competencies?
5	Assessing competence	Could you describe how staff competencies are assessed in your organisation?
		How systematic is the assessment of staff competence?
6	Managing a culture of competence	Could you describe the role of managers in the whole knowledge management process?
7	Other	Is there anything else you would like to add about knowledge management that has not been covered?

**Source(s):** The Authors’ own work

The interviews lasted on average 43 min (minimum 34 min, maximum 60 min). In total, the interviews amounted to 10 h and 49 min of recorded material. The sample size was considered adequate as data saturation had been reached, no new insights emerged, and redundancy was observed (Polit and Beck, 2017). With the manager's permission, all interviews were recorded and transcribed verbatim. The transcriptions resulted in a total of 165 pages, formatted in Calibri 11-point font with 1.15-line spacing. The transcriptions were not returned to the participants for comments or corrections.

### 2.6 Data analysis

A secondary qualitative analysis was conducted using an existing interview dataset that had originally been collected for a different research question and was subsequently reanalysed to address the aims of the present study (Polit and Beck, 2017). The data were analysed using deductive-inductive content analysis (Elo and Kyngäs, 2008). Deductive content analysis is appropriate when existing theory or prior knowledge informs the identification of key concepts within the data (Elo and Kyngäs, 2008), while inductive analysis is suitable when the phenomenon under investigation is not well explored or when existing knowledge is fragmented (Kyngäs *et al.*, 2020).

Deductive qualitative content analysis was first used to explore the phenomenon through an existing theoretical framework. The five dimensions of the Managers' Competence in Knowledge Management (MCKM) process model, previously developed and validated by the research group (Karsikas *et al.*, 2025a), served as the basis for the deductive analysis. These dimensions provided a structured lens for identifying and organising relevant content from the data concerning knowledge management in health and social care organisations.

The data were analysed using Microsoft Excel software. The analysis process began with the researcher becoming familiar with the interview transcripts through repeated reading and listening. Any theme or sentence that was relevant to the research question was defined as a unit of analysis. (Kyngäs *et al.*, 2020.) These original expressions were tabulated and simplified to capture their essential meaning. The simplified expressions were then deductively classified under the five predefined knowledge management dimensions from the MCKM process model.

Following the deductive classification, an inductive content analysis was conducted within each of the five dimensions. The expressions under each dimension were grouped into subcategories and further abstracted into main categories based on similarities and recurring patterns (Kyngäs *et al.*, 2020). These categories were named to reflect the underlying meanings emerging from the participants' perspectives. During the analysis process, the research team held regular meetings to discuss the coding, categorisation, and interpretation of the findings. They also worked together to validate the final analysis. Statistical measures, frequencies, percentages, means, standard deviations, and ranges were used to analyse the background data.

### 2.7 Rigor

The trustworthiness of the study was assessed according to the criteria of credibility, dependability, transferability, authenticity, and confirmability (Kyngäs *et al.*, 2020). Credibility was enhanced by selecting respondents according to pre-defined inclusion criteria and by achieving data saturation (Polit and Beck, 2017). Dependability was enhanced by the research group's participation in data collection and analysis. During the analysis phase, active discussions supported the integration of diverse perspectives and reduced individual bias. The provision of contextual information supported transferability. This allows the reader to determine whether the findings apply to other similar organisations and populations. Authenticity was enhanced using direct quotations. Confirmability was enhanced by clearly recording the progress of the research process.

### 2.8 Ethics

Research approval to conduct the study was obtained from each participating organisation (REDACTED). Vulnerable groups were not included in this study (World Medical Association, 2013). Participants were asked to provide informed consent. Participation was voluntary, and participants had the right to refuse to participate in the study or withdraw their consent at any time without penalty (World Medical Association, 2013). Participants' personal information was protected and kept confidential. Results were reported in a manner that allowed individual respondents to remain anonymous (Polit and Beck, 2017). All electronic data collected in the survey, including backup copies, was stored on a secure network drive under a user ID and appropriately destroyed after the results were reported.

## 3. Findings

### 3.1 Demographics

Of the participants (*n* = 15), 14 were women. Eight worked in public university hospitals and seven in public primary health and social care. The level of education varied, but the most common degree was a master's degree from a university, held by six participants. Most positions were first-line managers (53%). The mean age of the participants was 53.5 years, and they had a mean of 16.2 years of management experience in health and social care (see Table 3).

**Table 3:** Demographic profiles of participants (*N* = 15)

		Frequency	Percentage (%)
Gender	Female	14	93
	Male	1	7
Highest level of education	Master's degree	10	68
	Bachelor's degree	3	19
	Doctoral degree	2	13
Health and social care setting	Public university hospital	8	53
	Public primary health and social care	7	47
Levels of management	First-line manager	8	53
	Middle manager	6	40
	Top manager	1	7

	Mean	Standard deviation	Range
Age (years)	53.5	8.2	41.0–65.0
Management experience in health and social care (years)	16.2	9.2	5.0–35.0
Number of units to be managed	5.8	8.3	0–25
Number of direct staff	140	247.3	0–900

**Source(s):** The Authors’ own work

### 3.2 Perceptions of the knowledge management implementation in health and social organisations

For each of the five dimensions, anticipating and defining competence, planning competence development and cooperation, developing competence, assessing competence, and managing a culture of competence, managers' perceptions of knowledge management implementation in health and social care organisations were identified. A total of 398 simplified expressions emerged were identified in the data and systematically grouped into 56 sub-categories and 16 categories (see Table 4). Additionally, the results have been presented in a figure (see Figure 1).

**Table 4:** Implementing knowledge management in health and social care organisations according to managers

Anticipating and defining competence	Planning competence development and cooperation	Developing competence	Assessing competence	Managing a culture of competence
*Systematic anticipation of competence* No systematic anticipation No defined anticipation priorities in the organisation's strategy Competence anticipation depends on managers Different anticipation roles among managers *Data-based anticipatory competence* Analysis of demographic trends in society Follow national and international research and recommendations Using human resource management system data for competence anticipation *Anticipating competencies across career stages* Integrate competence anticipation into recruitment Improving competence anticipation through mentoring Addressing competence needs in development discussions *Collaborative approaches to competence anticipation* Internal meetings Using professional networks *Defining staff competence* Define competence for different professional groups Definition of disease-specific competencies No systematic definition	*Systematic competence development planning* Set clear priorities Consider employee readiness and competence gaps Lack of a competence development planning tool Absence of a strategic plan for both short-term and long-term competence development needs *Documentation of the competence development plan* Create a unit-specific competence development plan Create a unit-specific training plan Create a personalised competence development plan Create a personalised mentoring plan *Cooperation partners in planning competence development* Cooperation with universities Partner with professional associations Work with projects Engage with equipment suppliers Leverage the expertise of other units Use expert networks Managers to seek out competence development partners	*Internal competence development* Internal training Internal mentoring Co-development Collective working Simulations *Externally competence acquisition* External training Online courses and e-learning Specialist education through educational institutions	*Systematic assessing competence* Establish targets and monitor progress Evaluate adverse events and assess competence risks Address the lack of standardised competence assessment practices *Feedback and communication on competence* Collect and analyse customer feedback Communicate with staff on the state of competence Communicate with managers on the state of competence *Assessing competence at different career stages* Assessing competence during recruitment Assess competence throughout mentoring programs Integrate competence assessment into development discussions *Different methods and practices for assessing staff competencies* Medical authorisations Self-assessment survey	*Coaching leadership* Recognising staff potential Fostering a supportive community Mentoring and engaging in constructive sparring Delivering effective feedback Encouraging openness and shared accountability *Change management* Strategically prioritising input Taking decisive and goal-oriented actions

**Source(s):** The Authors’ own work

**Figure 1:**
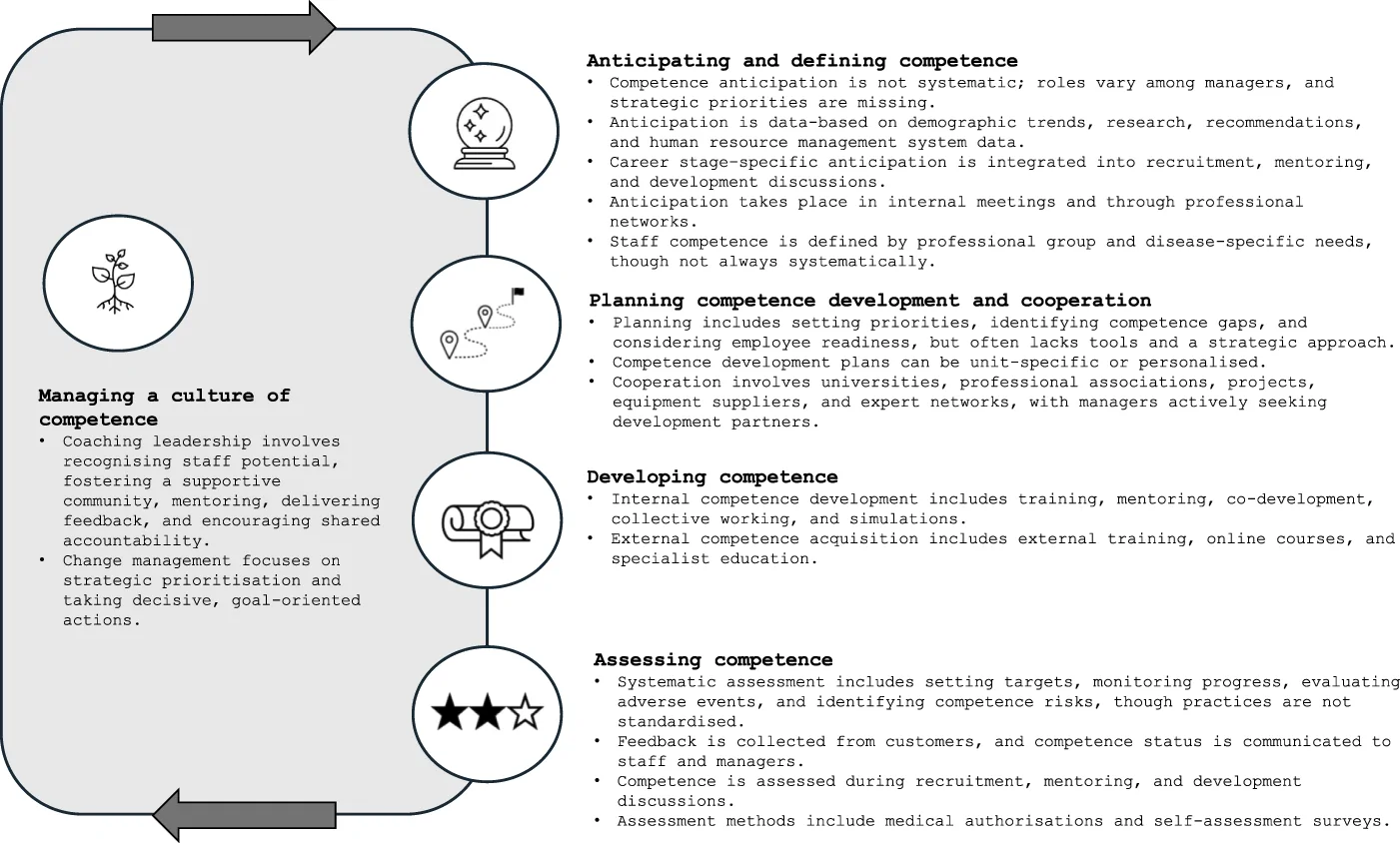
Implementing knowledge management in the different dimensions of the managers' competence in the knowledge management process model

#### 3.2.1 Anticipating and defining competence

The dimension of anticipation and definition of competence included five categories: systematic anticipation of competence, data-based anticipatory competence, anticipating competencies across career stages, collaborative approaches to competence anticipation, and defining staff competence. According to health and social care managers, the implementation of anticipation was limited by the lack of a systematic approach. Managers stated that organisations were often behind in terms of competence because they lacked a broader vision of anticipation. They also reported that the organisation's strategy had not defined competence priorities to guide the direction of anticipated work, and that competence management was anticipated to be manager and role-specific. Additionally, said:

Another manager insists that we do exactly what is said from the top, while another takes a different approach (emphasising anticipation). Manager, 10

And it’s precisely that impact, the fact that we probably need to make some adjustments in nursing management, that would make it (anticipate) more systematic, clearer, and something our nurses could also understand). Manager, 13

Health and social care managers emphasised the importance of utilising existing data sources to effectively anticipate future competence needs within organisations. They reported that organisations assess the state of competence by analysing demographic information (such as demographic trends, retirements, morbidity, and graduates) and national and international research and recommendations. In addition, managers incorporated organisational human resource management data — such as turnover, retirements, exits, and education and training data — to anticipate future competence needs. One manager explained:

Yes, well, at the organisational level we do, in a way, look at and anticipate retirements, and then anticipate … but I don’t really know how retirements, part-time work, sick leave, or similar factors actually influence training needs. I don’t really see how those are connected to identifying training needs. Manager, 11

According to the managers, competence anticipation is a continuous process that spans the entire employee lifecycle. It begins during recruitment, when managers assess the competencies, candidates may contribute to the organisation. The process continues during the onboarding phase, particularly through mentoring discussions with new employees. For more experienced staff, development discussions provide a forum for identifying future competence needs and formulating individual development plans:

When an employee or recruitment has taken place, and well, that competence anticipating it actually starts already from the recruitment, so their background is mapped, and then that orientation plan is tailored, like, at the unit level by the first-line manager. Manager, 5

In addition to these individual-level practices, competence anticipation also, and the use of professional networks occurs at the organisational level through internal meetings.

We have started new collaborative meetings of this kind to go through (anticipate) statistical data. Manager, 5

Managers reported that their organisations had defined competencies for managers, registered nurses, practical nurses, paramedics, physiotherapists, occupational therapists, radiographers and secretaries. Additionally, some organisations had defined competencies related to the care of specific patient groups, such as those with cardiac conditions, on-call needs, psychogeriatric conditions, palliative care needs, infections, or diabetes. However, managers pointed out that the competence of all staff at all levels of the organisation was not comprehensively defined. The reason for this, they said, was that top managers in the organisation had not given sufficient importance to this task:

There should be a plan on how we are going to implement it (define competence) and whether we are asking people what we are asking and at what level. Manager, 1

#### 3.2.2 Planning competence development and cooperation

The dimension of planning competence development and cooperation encompasses three categories: systematic competence development planning, documentation of the competence development plan, and cooperation partners in planning competence development. According to managers, planning for competence development in social and health care organisations involves setting clear priorities. Systematic planning considers staff readiness, supports continuous learning, and promotes the effective application of new competencies in practice. With a stronger emphasis on knowledge management implementation, managers highlighted a key challenge: many organisations lack the necessary tools to plan and document competence development. They noted difficulties in integrating different time horizons into the planning competence process:

Development plans that extend beyond a year, and similar, aren’t really in place … I can’t really say what the reasons for that are. Manager 4

Managers reported that documentation of planning competence development is accomplished through various types of plans. A unit-specific competence development plan is created to outline the broader knowledge management process for the unit as a whole. Additionally, a separate unit-specific training plan is developed to outline specific training actions. On a more individual level, a personalised competence development plan is prepared for existing staff to support their continued professional growth. For new staff, a personalised mentoring plan is developed to support their induction and integration into the unit.

In units, this is actually done quite well, even at the individual level, there is reflection and documentation as part of the development discussion, recorded in a personal document. Manager 7

Managers reported cooperation with various partners in planning competence development. These include universities, professional associations, and project partners. Equipment suppliers and expert networks are also utilised. Additionally, the expertise of other units is leveraged, and managers actively seek suitable development partners:

Well, I was thinking that it’s good to be an active player in building networks, and in a way, that enables collaboration by creating those structures. Manager 9

#### 3.2.3 Developing competence

The competence development dimension included two categories: internal competence development and external competence acquisition. Managers emphasise that both internal and external competence development strategies are essential and mutually reinforcing for the effective implementation of knowledge management. Managers highlighted that internal training was a key component and was offered to all staff, including both workers and managers. This training covered critical areas, including resuscitation and medication authorisation. Internal mentoring programs further strengthened organisational competence by facilitating knowledge transfer between experienced and less experienced employees, fostering continuous professional growth. Additionally, managers viewed co-development as a crucial approach for integrating knowledge management into daily operations. By involving different internal stakeholders in the design and refinement of services, processes, and policies, organisations ensured that knowledge was actively shared and applied in practice. Collaboration across teams was also seen as essential for disseminating knowledge and fostering a culture of collective learning. To enhance the development of practical competence, organisations use simulations tailored to their specific needs. These simulations helped employees practice and prepared for real-life situations effectively:

Yeah, well, we do have simulations, which have increased n in recent years, and I'm sure they'll keep increasing in many areas. Manager 7

Managers viewed off-the-job learning as a key tool for developing staff competence. They reported that organisations made extensive use of external training, online courses, and e-learning opportunities provided by external providers to broaden learning options. Additionally, organisations actively encouraged employees to pursue specialised training through educational institutions. Employees also took the initiative to seek such training themselves, aiming to deepen their expertise, acquire new competence, and advance their careers:

… well, I've set aside funds so that, for example, we currently have four nurses enrolled in a specialist education in acute care nursing. In previous years, we've had nurses enrolled in the on-call nurse program. So, I've set aside money. Manager 8

#### 3.2.4 Assessing competence

The assessing competence comprised four categories: systematic assessing competence, feedback and communication on competence, assessing competence at different career stages, and various methods and practices for assessing staff competencies. Health and social care managers viewed competence assessment as an essential component of implementing knowledge management, emphasising the need for clear objectives and systematic monitoring. Managers highlighted that assessments should specifically address reported adverse events and risks, as these often stem from insufficient competence. However, they also acknowledged that, in the absence of standardised practices, assessments were frequently based on subjective perceptions rather than objective, measurable indicators:

In the centre, we do not have that kind of–in my opinion, it has not even been defined how it (assessment) happens. Manager 2

Managers also emphasised the importance of feedback and communication in competence assessment. They view customer feedback and open dialogue with employees and managers as essential to the organisation's assessment of competence.

The quality of nursing care can be looked at from HaiPros (Reporting System for Safety Incidents in Health Care Organisations) at the moment, for example, and from customer feedback, like. Manager 3

Additionally, competence assessment is perceived as occurring at various career stages. It is integrated into the recruitment process by identifying the competencies that new employees bring to the organisation, as well as mentoring and annual career development discussions:

We do have personal development plans for staff, but at the managerial level this is largely absent. When I think about it now, I can’t really say much about it. When I was recruited and started working, my competence was assessed by asking me questions. Manager 1

Managers in the health and social care sector identified medical authorisations and self-assessment surveys as key methods for assessing staff competencies in organisations. Medical authorisations provide formal validation of professional qualifications and compliance with regulatory standards. Self-assessment surveys, in turn, serve as a reflective tool for staff to assess their competence and identify areas for development.

It's not exactly systematic, but there was some kind of little survey or something like that. Manager 6

#### 3.2.5 Managing a culture of competence

The dimension of managing a culture of competence encompassed two categories: coaching, leadership, and change management. Managers in health and social care organisations view knowledge management as closely tied to coaching leadership. They emphasise the importance of recognising employee potential and creating a supportive community. Through mentoring and constructive sparring, managers help employees refine their competence and provide feedback to ensure continuous learning aligned with organisational goals. Additionally, managers emphasise the importance of openness and shared accountability, as these elements enhance collaboration and facilitate the practical application of knowledge. By integrating these leadership practices, health and social care organisations can enhance their ability to adapt to evolving competence needs and ensure that knowledge management becomes an integral part of daily management:

It is sparring and support, making sure we are doing the right things, and then if it looks like we are not going in the right direction. Manager, 2

In health and social care organisations, managers view knowledge management as an integral part of change management, in which strategic prioritisation and goal-oriented decision-making are essential. According to managers, organisations sometimes need to make specific investments to achieve certain goals. In addition, managers' competence in planning and general management is crucial to the successful implementation of knowledge management.

Yes, it requires me as a manager to know what we want, what our goal is, where we are going, and what it takes. I need to have a vision and a vision for change. Manager, 12

## 4. Discussion

This study offers insights into how health and social care managers perceive the implementation of knowledge management within their organisations. The significance of this research is underscored by previous studies demonstrating that effective knowledge management, for example, is connected to the advancement of evidence-based practices in health and social care settings (Tajafari and Fanoodi, 2025) as well as to the overall performance of organisations (Karamitri *et al.*, 2020).

Based on our results, health and social care organisations anticipate competence needs at various stages of employees' careers. This practice is critical given that previous research has shown that about half of nurses consider leaving the profession (Taylor-Clarke *et al.*, 2022). Early identification of potential turnover is crucial to prevent competence loss, as high employee turnover can deplete critical expertise. In addition to these discussions, organisations monitor the number of graduates and the number of upcoming retirements across different professional groups. The effective use of such data is crucial for addressing future workforce shortages and ensuring the continuity of high-quality care. These findings are consistent with earlier studies highlighting the importance of strategic workforce planning in securing a competent and sustainable health workforce (Boniol *et al.*, 2022).

Our findings suggest that social and health care organisations may not yet have sufficiently systematic tools for planning staff competency development. This is despite earlier studies emphasising that various tools can improve the process of implementing knowledge management (Karamitri *et al.*, 2017). Furthermore, this finding aligns with previous research, which concluded that competence planning tools are not utilised as practical resources in daily work (Søreide *et al.*, 2019). Despite the lack of such tools, our findings indicate that personalised competence development plans are created within the organisation and discussed during development interviews. This observation aligns with previous research, which emphasises that career planning supports decision-making, career progression mapping, and actions that foster both personal and professional growth (Gunawan *et al.*, 2019). Special focus is needed to improve career development for substitute nurses, as they have been shown to have fewer opportunities for advancement than permanent nurses (Tan *et al.*, 2025).

Our results indicate that health and social care organisations seem to have established several approaches to develop staff competencies. This aligns with previous findings indicating that all organisations have processes for knowledge development (Kosklin *et al.*, 2022). For example, online courses and e-learning were regarded as effective methods for enhancing staff competence. This finding complements earlier research that has identified various knowledge management practices and methods in health and social care organisations but has not previously highlighted online learning as one of them (Karsikas *et al.*, 2025a). These practices demonstrate how knowledge management is implemented in practice through structured learning and development activities. Additionally, our findings indicate that the organisation seems to encourage staff participation in specialised training. This is a positive development, as previous studies have shown that healthcare organisations value the in-depth expertise of professionals with specialised training and often prioritise their recruitment (Lunden *et al.*, 2017).

Our results showed that organisations systematically assess staff competence through feedback, communication, medical authorisations and surveys. This finding contrasts with a previous study, which found that one in three nurses felt their competence was rarely evaluated or ensured by managers (Lunden *et al.*, 2021). Earlier research suggests that organisations tend to ensure nurses' competence in both theoretical knowledge and practical competence through regular dialogue and feedback, rather than relying solely on formal performance indicators (Kiviniitty *et al.*, 2023). Furthermore, our study highlights that competence is assessed during the recruitment process, with frontline managers actively evaluating candidates' competence. This supports previous findings on the use of competencies to compare nursing applicants (Gunawan *et al.*, 2019).

The findings of this study suggest the importance of recognising staff's potential for competence in implementing knowledge management and fostering a culture of competence. These findings align with previous research by Søreide *et al.* (2019). Their study suggested that improving services does not necessarily require additional competence but rather the reorganisation of existing competence. By maximising the creation, accessibility, sharing, and application of relevant knowledge through the effective utilisation of staff potential, organisations can optimise existing competencies to enhance service delivery and overall performance (Ayatollahi and Zeraatkar, 2020; Søreide *et al.*, 2019). Additionally, our study highlighted the significance of constructive sparring with employees as a method for implementing knowledge management but did not mention rewards.

Although the results were organised according to the five dimensions of the MCKM framework, we recognise that adopting a predefined analytical structure may limit the emergence of findings outside the framework. While efforts were made to remain open to the data, the analysis was nevertheless guided by the MCKM dimensions from an early stage. Importantly, the purpose of this study was not to revalidate the MCKM process model, which has previously been validated as a measurement instrument, but to apply it as an analytical lens (Karsikas *et al.*, 2025b). The analysis, therefore, illustrates how managers' perceptions of knowledge management processes and related activities correspond to the predefined dimensions, rather than how these activities are systematically operationalised within their organisations. All simplified expressions identified in the analysis could be classified within the five dimensions, and no additional dimensions emerged from the data. This outcome reflects the nature of the data, which captures managers' perceptions and interpretations of knowledge management practices. Accordingly, the findings represent perceived implementation rather than verified organisational routines. At the same time, the strong alignment between managers' descriptions and the MCKM dimensions may suggest the framework's conceptual resonance in this context.

Future research should employ study designs and data specifically developed to examine knowledge management implementation. Further studies should include managers from different organisational levels, particularly senior leadership, and ensure broader representation of social care organisations. Comparative research between health and social care settings, as well as public and private sector organisations, is needed to better understand contextual influences on knowledge management implementation. In addition, studies conducted in diverse geographical and organisational contexts would strengthen the transferability of findings and provide a more comprehensive understanding of knowledge management practices. It is also essential to assess the effectiveness of new technology integration, particularly the role of artificial intelligence and machine learning, in enhancing knowledge management processes. Furthermore, knowledge management implementation should be studied and developed from an interdisciplinary perspective (Stoumpos *et al.*, 2024).

## 5. Limitations

Although the study provided valuable insights, it also had several limitations. One key limitation was that the interview questions were not initially designed to address the specific research questions of this secondary qualitative analysis. Additionally, the researchers' backgrounds (e.g. professional experience in health and social care, prior clinical or managerial work, educational background) and potential biases may have influenced the data analysis and, consequently, the findings. The study's geographical scope also limits the generalizability of the results to other contexts. The gender distribution and limited representation of senior management, with only one senior manager participating, may have impacted the results. As more than half of the respondents were first-line managers, the findings may predominantly reflect the operational level's perspective on knowledge management implementation. Nevertheless, the sample can be considered adequate, as it includes individuals with more than five years of experience in management positions.

Most participants worked in health care organisations, which may mean that practices used in social care settings are not fully represented. Therefore, caution should be exercised when applying the findings directly to social care organisations. This study was conducted in the public social and healthcare sector, which may yield findings different from those in the private sector. However, previous research suggests that the gap in human resource management practices between the public and private sectors is narrowing (Knies *et al.*, 2022). Another notable limitation is that the interviews were conducted via Microsoft Teams. The remote format may have affected the quality of the data by limiting the richness of face-to-face interaction.

## 6. Conclusions

The five dimensions of knowledge management provided a valuable framework for gaining deeper insight into how it is implemented in health and social care organisations. Knowledge management implemented within the organisation demonstrates a partially structured and systematic approach. Elements such as competence development plans and assessments are carried out to some extent in an organised manner. However, key gaps remain, including the absence of systematic anticipation, strategic priorities, planning tools, and standardised assessment practices. This structured foundation supports long-term knowledge management across different career stages, where both the anticipation and assessment of competence are aligned with professional growth and organisational objectives. Additionally, cooperation is crucial to this process, involving joint efforts in anticipating future competence needs, planning development activities with partners, and engaging in collaborative work practices and cooperation with educational institutions. Coaching-oriented leadership and effective change management are key enablers of implementing sustainable knowledge management in practice, supporting both individual growth and organisational adaptability.

### 6.1 Implications

#### 6.1.1 Implications for theory

This study contributes to the knowledge management literature by providing an empirical description of how knowledge management is implemented in health and social care organisations across different stages of the knowledge management process. The findings suggest that knowledge management should be understood as a comprehensive and interconnected process rather than as a collection of separate practices. Although organisations use a wide range of knowledge management methods, their application may vary and lack coherence, highlighting the need for further theoretical development on integrating the different dimensions of knowledge management in organisational practice.

#### 6.1.2 Implications for practice

The results can be used to strengthen knowledge management practices in health and social care organisations. For example, organisations may benefit from developing more structured tools and processes to plan and monitor staff competence development. The findings also emphasise the importance of robust implementation and suggest that knowledge management should be approached as a comprehensive strategic process capable of improving organisational effectiveness. In the short term, organisations may benefit from better coordination and integration of existing practices. In the longer term, knowledge management should be more strongly embedded in strategic planning, decision-making, and everyday management processes. In addition, the findings may help guide the education and training of managers by strengthening their competence in implementing knowledge management.

#### 6.1.3 Implications for policy

Strengthening knowledge management requires more systematic and clearly defined actions to enhance the attractiveness and sustainability of the public sector workforce. As health and social care systems are largely government-driven, the findings highlight the importance of strategic prioritisation and policy-level guidance in supporting knowledge management implementation. Policymakers should recognise knowledge management as a key enabler of competence development, organisational learning, and workforce retention while taking into account public-sector constraints that may affect implementation.
